# Using Artificial Intelligence Tools as Second Reviewers for Data Extraction in Systematic Reviews: A Performance Comparison of Two AI Tools Against Human Reviewers

**DOI:** 10.1002/cesm.70036

**Published:** 2025-07-14

**Authors:** T. Helms Andersen, T. M. Marcussen, A. D. Termannsen, T. W. H. Lawaetz, O. Nørgaard

**Affiliations:** ^1^ Copenhagen University Hospital—Steno Diabetes Center Copenhagen Herlev Denmark

**Keywords:** artificial intelligence, ChatGPT, data extraction, Elicit, large language models, research methodology, systematic review methodology

## Abstract

**Background:**

Systematic reviews are essential but time‐consuming and expensive. Large language models (LLMs) and artificial intelligence (AI) tools could potentially automate data extraction, but no comprehensive workflow has been tested for different review types.

**Objective:**

To evaluate Elicit's and ChatGPT's abilities to extract data from journal articles as a replacement for one of two human data extractors in systematic reviews.

**Methods:**

Human‐extracted data from three systematic reviews (30 articles in total) was compared to data extracted by Elicit and ChatGPT. The AI tools extracted population characteristics, study design, and review‐specific variables. Performance metrics were calculated against human double‐extracted data as the gold standard, followed by a detailed error analysis.

**Results:**

Precision, recall and F1‐score were all 92% for Elicit and 91%, 89% and 90% for ChatGPT. Recall was highest for study design (Elicit: 100%; ChatGPT: 90%) and population characteristics (Elicit: 100%; ChatGPT: 97%), while review‐specific variables achieved 77% in Elicit and 80% in ChatGPT. Elicit had four instances of confabulation while ChatGPT had three. There was no significant difference between the two AI tools' performance (recall difference: 3.3% points, 95% CI: –5.2%–11.9%, *p* = 0.445).

**Conclusion:**

AI tools demonstrated high and similar performance in data extraction compared to human reviewers, particularly for standardized variables. Error analysis revealed confabulations in 4% of data points. We propose adopting AI‐assisted extraction to replace the second human extractor, with the second human instead focusing on reconciling discrepancies between AI and the primary human extractor.

## Summary

- What This Study Is About
  ◦ This study tested if AI tools (Elicit and ChatGPT) could help speed up systematic reviews by pulling out important information from research papers, potentially replacing one of the two human reviewers usually needed.
  ◦ AI tools are software applications that use artificial intelligence algorithms to perform tasks that require human intelligence.
- What They Did
  ◦ The researchers compared how well the AI tools could extract specific information from 30 research articles vs. how human reviewers did it.
  ◦ They looked at things like study details, participant information, and other key data.
- What They Found
  ◦ Both AI tools extracted the right data about 9 out of 10 times.
  ◦ The AI tools were best at identifying basic study information and participant details.
  ◦ The AI tools sometimes made‐up information (3%–4% of the time). Elicit and ChatGPT performed similarly well.
- What This Means
  ◦ Instead of having two people extract data from each paper, researchers could have one person, and one AI tool do the initial review, with another human checking any differences.
  ◦ This could make systematic reviews faster and cheaper while maintaining quality

## 1 Introduction

Systematic reviews play a key role for evidence‐based decision‐making, carefully summarizing existing information on specific topics. The process of conducting systematic reviews is time‐consuming and labor‐intensive with a mean of 67 weeks but up to 2 years of work [1, 2]. Further, an estimated average expense of more than $140,000 per review and an average yearly cost of more than $18,000,000 per academic institution calls for the involvement on machine learning/artificial intelligence (AI) to aid the promise of evidence‐informed decision making [3].

Data extraction for systematic reviews—one of the most time‐consuming but critically important tasks, entails at least two humans extracting the same data, subsequently comparing and reconciling what was extracted [1, 4]. Large language models (LLMs) are changing the landscape for data synthesis, and several studies have evaluated the use of LLMs to automate data extraction and speed up the process as well as reduce human errors [5, 6, 7, 8, 9, 10].

To our knowledge, no one has yet tested and compared a publicly available, comprehensive workflow with prompts adaptable to different review types. Therefore, the objective of this study was to evaluate Elicit's and ChatGPT's abilities to extract data from journal articles reporting quantitative data from primary studies to replace one of the two human data extractors in systematic reviews.

## 2 Terminology

In AI research, multiple terms including “hallucination,” “error,” and “confabulation” are used to describe inaccurate outputs from LLMs. We adopt “confabulation” in this article, when models output data that has no basis in the data presented to it, and “error” when it handles, presents or omits data in a misleading manner. The term “hallucination” incorrectly implies sensory perception and consciousness that AI does not possess, where the term “confabulation” more accurately describes the generation of fabricated but “logically generated semantic statements” based on statistical patterns in training data [11]. This terminology, borrowed from neuropsychology, provides a more precise conceptual framework for understanding LLM inaccuracies and helps prevent unintentional stigmatization of both AI systems and persons who experience actual hallucinations [12].

## 3 Materials and Methods

**Figure 1:**
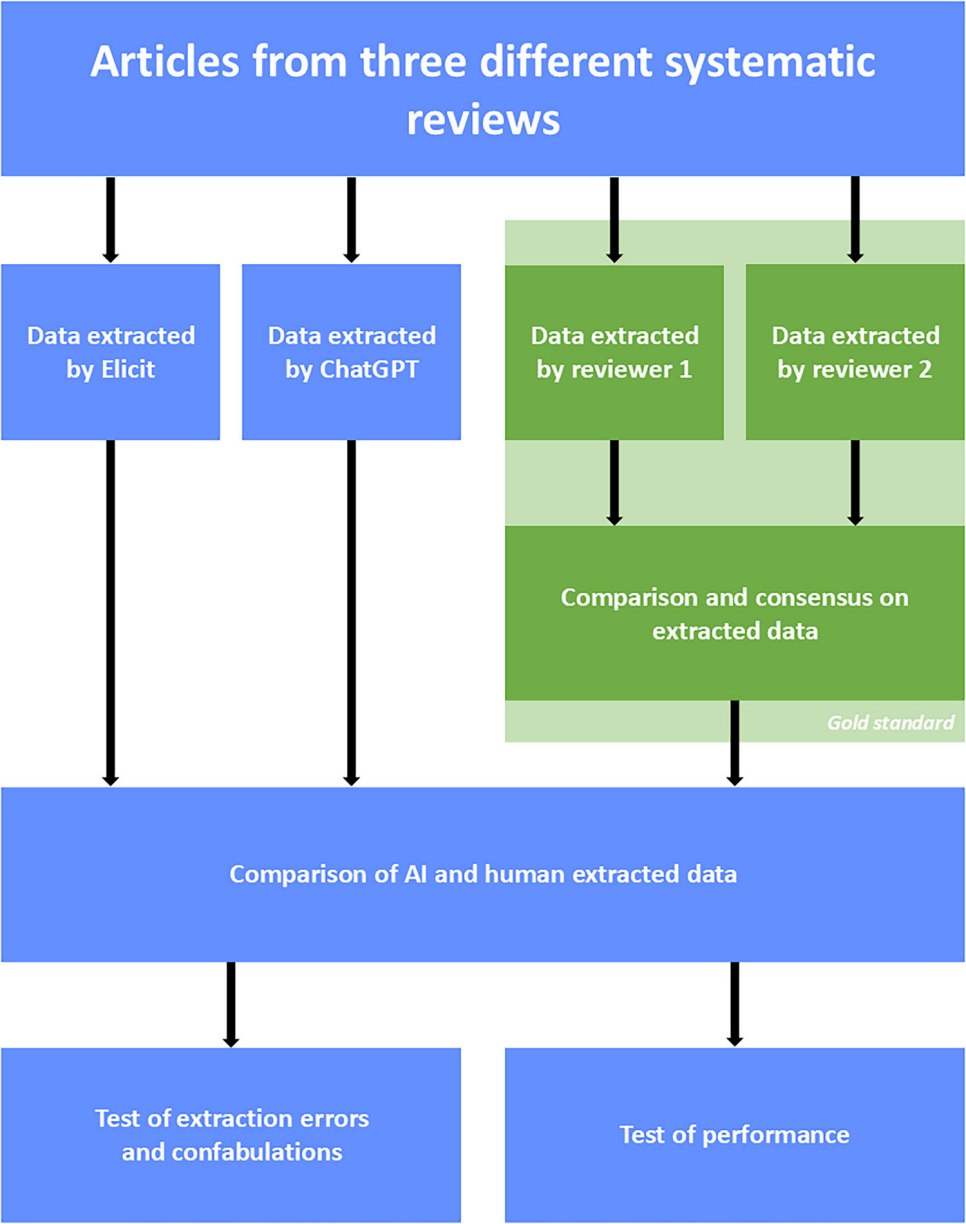
Diagram of the study design. The green boxes represent the gold standard performed independently in the three systematic reviews. The blue boxes represent the processes performed in the present study. Data from a random subset of the original articles was extracted using both Elicit and ChatGPT.

Human‐extracted data from articles included in three different types of systematic reviews, Andersen et al. [13], Lawaetz et al. [14] and Termannsen et al. [15] was compared to data extracted by Elicit [16] and ChatGPT [17]. The AI tools' performances were compared to human double‐extracted data as the gold standard. A study design diagram is shown in Figure 1.

### 3.1 Selection of AI Tools

Elicit was chosen because it entails a rapid, streamlined workflow in which all included full‐text articles can be uploaded simultaneously. The prompts used in Elicit can also be easily modified and applied to all articles at once. Further, all extracted data can be exported in CSV format, facilitating straightforward comparison with human‐extracted data.

ChatGPT was chosen because it is a widely recognized chatbot that many researchers are familiar with, making it an easy go‐to option for smaller sets of included articles and data items. In this study, we used ChatGPT's GPT‐4o model (version gpt‐4o‐2024‐05‐13), henceforth referred to as “ChatGPT,” for prompting.

Data was extracted on 5 July 2024 in both tools.

### 3.2 Selection of Studies

The systematic reviews include 10, 11, and 39 studies, respectively. The three reviews represent numerical and text‐based data formats from both interventional and non‐interventional studies. For the two reviews containing more than 10 studies, a random selection of 10 articles from each systematic review was obtained using the sample function in R software (version 2024.04.2 + 764) [18]. Articles were included for randomization if they were available as searchable PDFs and excluded if they were image‐only or scanned PDFs. For the review by Andersen et al., sampling was performed solely on studies based on quantitative data.

### 3.3 Prompting and Extracting Data With AI

All prompts used in this study consisted of two parts: a prefix and a task‐specific prompt. The prefix prompt instructed the AI tool to take on the role of a reviewer and to adhere to strict, systematic extraction rules. This was followed by the task‐specific prompt. All prompts were tested on five sample articles and refined in four iterations by three authors (TMM, THA and ON).

We designed three task‐specific prompts to evaluate the ability of Elicit and ChatGPT to identify population characteristics, study design and a “review‐specific variable” for the primary outcome of the review. All prompts were pre‐defined, but those focusing on population characteristics and the review‐specific variable include modifiable parts, allowing for easy customization and extraction of the required information.

All prompts are made available to use by future researchers in Appendix 1, with square brackets indicating the parts of each prompt that reviewers must customize to obtain the variable(s) that is relevant for their review.

Elicit was instructed to use its built‐in “high accuracy” mode, and the prompt was inserted into the instructions section of three separate custom columns.

Using ChatGPT, we started a new chat for every article. Within one chat, all three prompts (including prefix and task‐specific prompt) were performed as three consecutive prompts. The article PDF was only uploaded alongside the first prompt.

### 3.4 Human Data Extraction

The human data extraction was conducted before and independently of the initiation of this study and was initially performed by two researchers independently. For the present study, this human‐extracted data served as a gold standard against which the AI‐extracted data was compared.

All the human‐extracted data is published in peer‐reviewed journals.

### 3.5 Comparison

Each data item was compared to the human data extraction by the original authors of the systematic reviews. A researcher not affiliated with the review validated this comparison. Any discrepancies were resolved by involving a third researcher. Finally, one author read all comparisons to assess consistency.

The AI‐extracted data was compared to the validated human‐extracted data using the questions in Table 1.

**Table 1:** Questions for comparing AI data extraction performance against human‐validated extractions.

1. Was the relevant data found? (data extracted and validated by humans)	2. Was data found that was irrelevant to the prompt? (data other than what was extracted and validated by humans)
1. All relevant data was found 1. Relevant data was found, but one data point was missing 1. Relevant data was found, but more than one data point was missing 1. Relevant data was found, but some was incorrect 1. Data that was incorrect was found 1. No data was found	2. Data was found that is in the primary article but does not match what was requested 2. No additional data

In four cases PlotDigitizer [19], a tool to extract data from graphs, had been used by the human extractors. All four cases were from the review by Lawaetz et al. [14] and relevant only to the review‐specific prompt. As the AI tools were not prompted to analyze or extract data from graphs, these cases were rated as “all relevant data was found” if all data except for the data given solely by PlotDigitizer was reported.

### 3.6 Analysis

To evaluate the performance differences between the two AI tools for data extraction, we employed both standard performance metrics and statistical significance testing. For each tool, we calculated precision, recall, and F1 scores to assess overall extraction quality.

To determine the performance and statistical significance of the difference between Elicit and ChatGPT, we conducted two‐proportion tests for precision and recall. Results are presented as percentages and with 95% confidence intervals (CI). Statistical significance was set at *p* < 0.05, two‐tailed. While we report the numerical difference in F1 scores, we did not perform statistical testing on this metric as the harmonic mean of precision and recall does not directly satisfy the assumptions required for proportion tests.

Additionally, we applied McNemar's test to examine the performance on a case‐by‐case basis focusing on the discordant pairs. To quantify the magnitude and direction of differences in the discordant pairs, we calculated the odds ratio with 95% confidence intervals (Table 2).

All statistical analyses were performed using R software [18].

To calculate precision, recall and F1, the six categories outlined in Table 1 were divided into true positives (TP), false positives (FP), and false negatives (FN). As we were working with a data set where all data points included relevant data, there were no true negatives (TN). Category 1a was labelled as TP, while categories 1d and 1e were labelled as FP because the data was extracted but contained confabulations. Extracts in category 1b, which represent mild underreporting, were labelled as TP, as such cases pose only minor inconvenience to the comparator, requiring confirmation of a single data point already obtained by the human extractor. Category 1c represents severe underreporting with little or no benefit from using AI and was labelled as FP. Category 1f was labelled as FN as it represents cases where no data was found even though we know it exist.

All measurements were rounded to the nearest integer to enhance data presentation while maintaining statistical relevance.

To further elucidate on the pattern of extraction errors we conducted a thorough examination of all errors while looking for patterns, error types, and differences between AI tools.

## 4 Results

After random selection, the total number of articles analyzed was 30, making up 180 unique AI‐extracted data points. The analyzed studies were published between 2005 and 2022. All studies included in the comparisons are listed in Appendix 2.

### 4.1 Elicit

For Elicit, all relevant data was found in 80/90 extractions and in 3/90 some relevant, but one data point was missing. In 3/90 some relevant but more than one data point was missing. In 3/90 some relevant but also some incorrect were found, and in 1/90 the extracted data was incorrect. Elicit had no cases of missing data extraction.

When extracting population characteristics 28/30 extractions included all relevant data and 2/30 included relevant but one missing data point. For study design all 30 extractions included all relevant data. When extracting the review‐specific variable all relevant data was found in 22/30 extractions and in one case relevant data was found but one data point was missing. In 3/30 relevant data was found but more than one data point was missing. In 3/30 cases relevant but also incorrect data was found and in one case only incorrect data was found.

### 4.2 ChatGPT

For ChatGPT, all relevant data was found in 76/90 extractions and in 4/90 some relevant but one data point was missing. In 5/90 some relevant but more than one data point was missing. In 2/90 some relevant but also some incorrect were found, and in 1/90 the extracted data was incorrect. In 2/90 no data was found.

**Figure 2:**
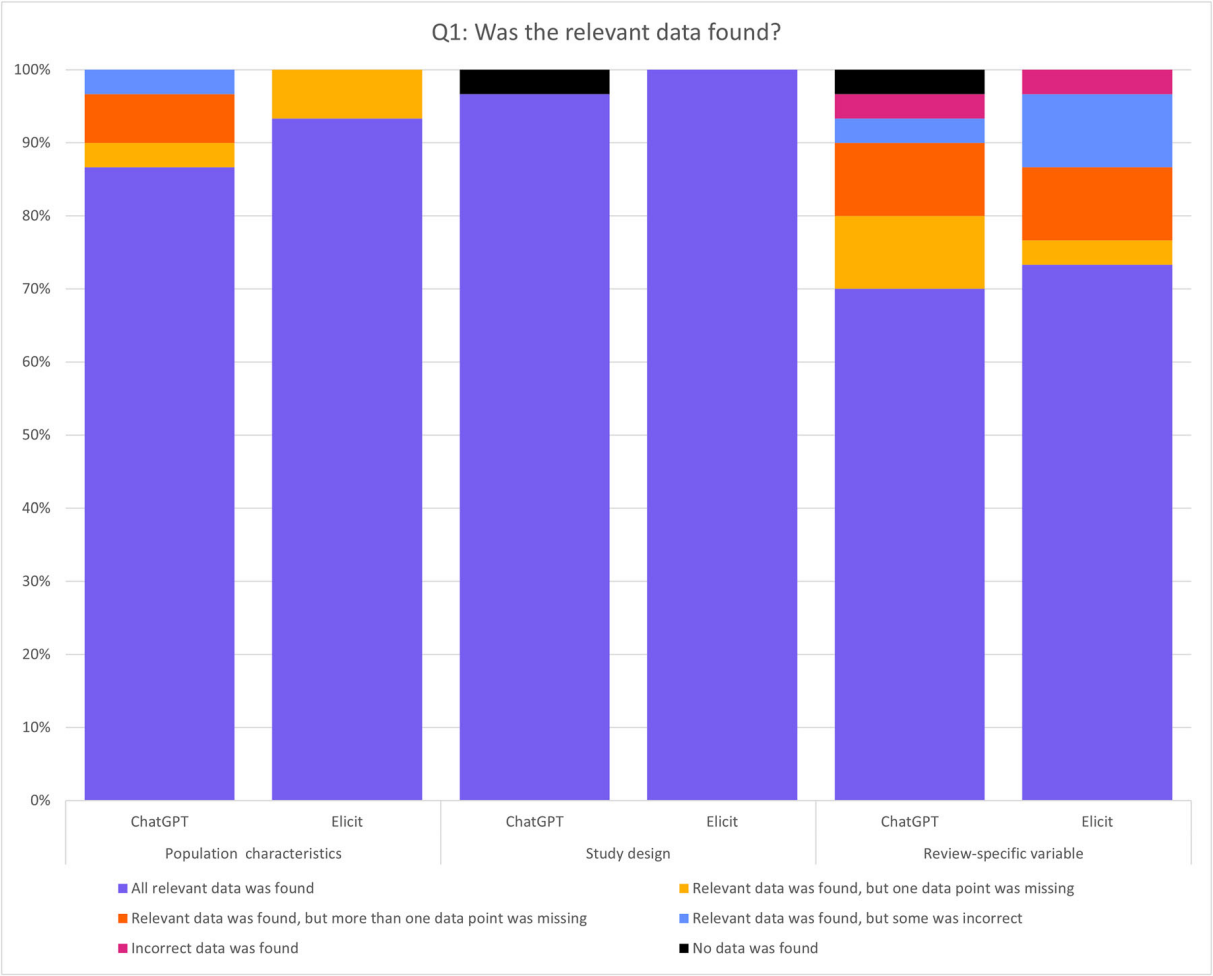
Distribution of data retrieval outcomes across population characteristics, study design, and review‐specific variable, showing the percentage of cases where all relevant data was found, partially missing, incorrect, or not found at all.

For the population characteristics 26/30 contained all relevant data, 1/30 had relevant data but one missing data point, 2/30 had relevant data but more than one missing and 1/30 had relevant but also incorrect data. Extractions of the study design variable showed all relevant data in 29/30 extractions and in one case no data were extracted. Extractions for the review‐specific variable found all relevant data in 21/30 cases and in 3/30 relevant data but one missing data point was found. In 3/30 relevant data was found but more than one data point was missing. In 1/30 relevant but also incorrect data was found and in 1/30 only incorrect data was found. In 1/30 no data was found. The distribution of data is shown in Figure 2.

### 4.3 Irrelevant Data

Data that was irrelevant to the prompt (cf. question 2) was reported in 3/180 (2%) of the extractions.

### 4.4 Comparative Analysis of Elicit and ChatGPT

Elicit and ChatGPT demonstrated similar performance across all metrics. For precision, Elicit achieved 92.2% compared to ChatGPT's 90.9%, a difference of 1.3% points. In terms of recall, Elicit correctly extracted 92.2% of the relevant data points, while ChatGPT extracted 88.8%, representing a difference of 3.3% points. The F1 scores, which provide a balanced measure of precision and recall, were 92.2% for Elicit and 89.9% for ChatGPT, a difference of 2.3% points (Table 2).

**Table 2:** Precision, recall, F1 score and test statistics comparing Elicit to ChatGPT.

Metric	Elicit	ChatGPT	Difference	95% CI	*p* value
Precision	92.2%	90.9%	1.3%	−6.9%–9.5%	0.753
Recall	92.2%	88.8%	3.3%	−5.2%–11.9%	0.445
F1 Score	92.2%	89.9%	2.3%		

The difference between the precision and recall for Elicit and ChatGPT was not statistically significant (precision: *p* = 0.753, 95% CI: −6.9%–9.5%; recall: *p* = 0.445, 95% CI: −5.2%–11.9%).

Table 1 presents a detailed comparison of both systems across all performance metrics.

### 4.5 Extraction Error and Confabulation Types

Analyzing the 24 extraction errors (questions 1b–f) with McNemar's test revealed no statistically significant difference in extraction errors between ChatGPT and Elicit (*p* = 0.45, OR = 1.67, 95% CI: 0.61%–4.54%). Error distribution analysis demonstrated minimal overlap between AI tools, with only one identical error observed (omission of change scores during extraction of the review‐specific variable for Barnard et al. [20]). The remaining 23 extraction errors were distributed as 10 errors unique to ChatGPT, six unique to Elicit, and three extraction points where both tools erred, albeit with distinct error typologies.

Error pattern analysis revealed a significant association between AI tool and error context. ChatGPT's unique errors predominantly occurred when extracting data from continuous text (70.0%), whereas Elicit's unique errors were primarily associated with tabular data extraction (66.0%). All cases where both tools produced errors at identical extraction points involved tabular data exclusively. Randomized controlled trials (RCTs) constituted 50.0% of unique errors for both ChatGPT and Elicit, and the identical error shared between the two tools. The Review‐specific variable presented the greatest extraction challenge of the variables, representing 50.0% of ChatGPT's unique errors, 66.6% of Elicit's unique errors, and the shared error.

We found no meaningful patterns for article length and extraction errors for either tool, with nearly identical average page counts for studies with and without errors. Document density (tables or media per page) showed no meaningful impact on error rates. High amounts of media elements did not influence error occurrence for either tool.

In cases where incorrect data (confabulations, question 1e and 1d) was found, we examined the types of confabulations occurring. Random value confabulations occurred when AI tools fabricated entirely fictitious data points that could not be found in the article provided. Incorrect labeling confabulations happened when AI tools reported a data point that could be found in the article, but labelled it with an incorrect name. Miscalculation confabulations involved the AI tool performing the correct type of calculation for the data point, but based it on the wrong values from the article. Rounding confabulations occurred when the AI tool reported a correct numerical data point but rounded it to the nearest whole number. Elicit had four (4%) cases of confabulation and ChatGPT had three (3%) out of 90 cases each. The two‐proportion z‐test yielded a cumulative difference of 1.11% points (95% CI: −4.54%–6.76%, *p* = 0.6998), again, indicating no significant difference between them. An overview of the distribution of confabulation types is shown in Table 3.

**Table 3:** Confabulation types stratified by AI tool. Random values: reporting of values that are not present anywhere in the PDF. Incorrect labelling: reporting values that are present in the PDF but labelled incorrectly. Miscalculations: reporting a correctly computed value, based on incorrect data. Rounding values: reporting values that are labelled correctly but rounded.

	Random values	Incorrect labelling	Miscalculations	Random values and rounding values	Random values and incorrect labelling	Total
Elicit	2	1	0	1	0	4
ChatGPT	1	0	1	0	1	3

## 5 Discussion

Both AI tools demonstrated a similar performance with precision, recall and F1 scores around 90%. Although Elicit demonstrated a slightly better numerical performance across all metrics compared to ChatGPT, the absence of statistical significance suggests that these differences may be attributed to chance variation rather than fundamental performance disparities between the two tools. All 24 extraction errors were analyzed to assess the differences in the tools' ability to extract data from continuous text and tables. Elicit had four instances of confabulations with ChatGPT having three. Notably, high accuracy rates were observed for population characteristics (Elicit: 100%; ChatGPT: 90%) and study design variables (Elicit: 100%; ChatGPT: 97%), while review‐specific variables achieved 77% in Elicit and 80% in ChatGPT. These findings provide a detailed empirical foundation for evaluating the practical value of AI tools in evidence synthesis workflows.

Our study demonstrates that AI‐based systems offer promising solutions for automated data extraction from scientific literature. The ability to rapidly process large volumes of published research addresses a critical bottleneck in knowledge synthesis across disciplines. Our findings align with recent advances showing that AI‐powered data extraction tools are progressively transforming evidence synthesis, offering substantial time and resource savings while maintaining accuracy [6, 21, 22]. Given the rapidly changing landscape of automating systematic review tasks, researchers have chosen different approaches to evaluate and test AI tools for data extraction. Khan et al. [23] and Konet et al. [9] evaluated two LLM models against each other with Kahn et al. testing a setup with double extracting data, mimicking the real world's two reviewer process obtaining reasonable performance. Gartlehner et al. and Khraishna et al. evaluated the performance of a single LLM and reported acceptable to high accuracy [6, 22]. While all of them achieved reasonable results they all highlighted extraction errors and emphasized the need for human validation. Our study evaluated adaptable prompts in two different easily assessable AI tools, with the possibility to create a practical workflow for implementation. We tested it on different study types and with a detailed focus on error types and confabulation types, altogether contributing to this important scientific field.

Beyond performance metrics, it is essential to consider the broader systemic effects of integrating AI into evidence synthesis workflows. The widespread use of AI‐assisted tools may not only deliver direct efficiency gains but also reshape the scientific information ecosystem in more far‐reaching ways. The integration of AI tools, such as Elicit and ChatGPT, has the potential to significantly accelerate the systematic review process by automating tasks like screening and data extraction [24]. This increased efficiency may lead to a higher volume of systematic reviews, raising concerns about redundancy and the pressure it places on the production of new primary research [25]. While this increased output may improve access to synthesized evidence, it may also shift scholarly incentives toward secondary analysis and away from original research. This dynamic is further complicated by findings that inclusion in systematic reviews can reduce citations to the original articles, potentially disadvantaging researchers whose work is incorporated but not directly acknowledged [26, 27]. Furthermore, the growing volume of evidence syntheses may overwhelm institutions responsible for translating research into clinical guidelines or policy, especially when multiple reviews address similar questions with varying conclusions [28]. While AI may eventually assist in synthesizing across such reviews, expert interpretation and domain‐specific judgment will remain essential to ensure reliability and contextual appropriateness [24]. These trends highlight the need for intentional governance and expert oversight to ensure that AI‐enhanced evidence synthesis strengthens—rather than distorts—the scientific information ecosystem. A key consideration in this governance is how AI can be responsibly integrated into specific stages in evidence synthesis workflows, such as data extraction, without compromising accuracy or scholarly integrity.

**Figure 3:**
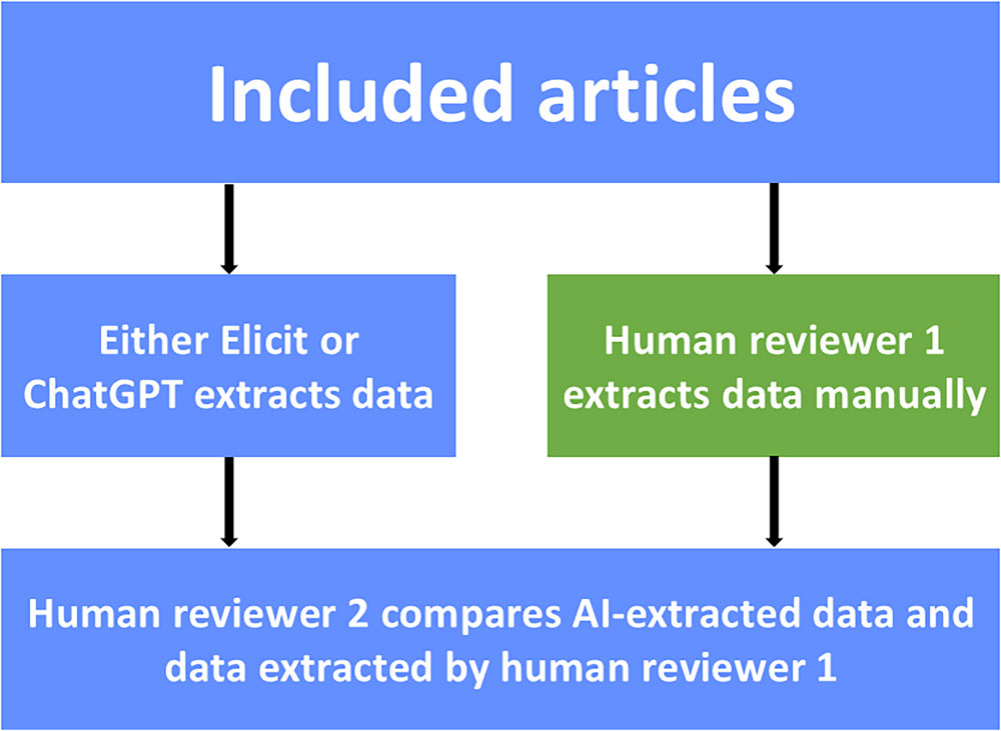
Proposed workflow for future application of AI tools in the data extraction process for systematic reviews.

Current systematic review methodology recommends independent dual extraction with subsequent reconciliation [4]. However, in practice, single extraction with verification by a second reviewer is frequently employed. Research suggests that this verification process may not always be conducted with sufficient rigor, and AI‐extracted data from our evaluation appears sufficiently accurate to potentially match one of the human reviewers with single extraction shown to have an error rate of more than 15% [29, 30]. However, both AI tools had extraction errors including confabulations, and we provide a detailed overview of the error and confabulation types. Though some patterns emerged in our analysis, errors and confabulations are diverse and rarely overlap between tools. These findings suggest that while AI tools may provide high‐accuracy data extraction similar to human reviewers and potentially streamlining the extraction and comparison phase, human‐first extraction remains crucial to mitigate the risk of incorporating confabulated data. We propose a modified workflow where the second reviewer would focus exclusively on reconciling discrepancies between human‐ and AI‐conducted extractions, potentially improving efficiency while maintaining high accuracy standards. This workflow is exemplified in Figure 3. The data extraction would remain highly accurate, and the process would save time and resources. This approach could be particularly valuable for reviews involving extensive data extraction or a large number of included studies.

In considering the broader applicability of these tools, cost and accessibility are important practical factors. At the time of our study, Elicit operated on a model where usage was based on prepaid tokens (credits). However, shortly thereafter, on 25 July 2024 [31], Elicit transitioned to a subscription‐based model. As of now, tasks of the scale conducted in this study can be performed using Elicit for a monthly fee of USD 12. For ChatGPT, we employed the Plus subscription, which costs USD 20 per month. Since 13 May 2024 [32], the GPT‐4o model has been made available for free, with a limited number of messages available to users. However, given our usage patterns, similar data extraction tasks would likely exceed the free message quota, necessitating a subscription comparable to ChatGPT Plus. These developments suggest that AI‐assisted extraction is not only accurate and efficient, but increasingly affordable and accessible to a broad range of users.

However, broader implementation considerations, including trust in automated processes and technical implementation challenges, remain significant barriers to adoption of AI in evidence synthesis workflows [33]. This call for a future focus on usability of AI in evidence synthesis [7] and our evaluation setup with adaptable prompts to use in different AI tools fits into building knowledge in that area. Given comparable performance between AI tools, platform selection should be based on review‐specific requirements and workflow preferences, with Elicit offering extraction from many articles at once, whereas ChatGPT offers a familiar and accessible interface for most users. Taken together, our findings contribute to the growing body of evidence supporting responsible, context‐specific integration of AI tools into systematic review practice.

### 5.1 Strengths and Limitations

The prompt development process followed a systematic and comprehensive methodology, with all prompts made publicly accessible, making it easier to copy our workflow. Further, quality control measures included multiple inter‐reviewer consistency checks during the comparison phase. The present study tested extraction of multiple data formats, with minimal extraction differences supporting generalized use across review types. The prompt's performance may vary when applied to entirely novel review types or articles with structures different from those included in this study, as our sample may not capture the full range of complexity found in systematic reviews across all disciplines.

A key limitation of our study is that we could not assess how these tools perform when information is genuinely unavailable (true negatives). To properly evaluate this aspect, a different experimental design that deliberately includes cases where the correct answer is “information not available” is needed. For each data point we only retrieved the AI answers once, instead of retrieving multiple answers and comparing each AI tool to itself. This entails a missing analysis of consistency within each AI tool. Our objective though, was to compare AI to a second reviewer within an easy workflow which can be used by most reviewers.

The changing landscape of LLMs and AI tools coupled with non‐transparent AI algorithms presents inherent limitations in generalizing our proposed prompting methodology. Though, until now, AI tools for research purposes have only gotten more precise, and with a human extraction first approach we believe that strict quality measures can be maintained.

## 6 Conclusion

Overall data extraction conducted by two AI tools in this study showed a similar performance of between 88.8 and 92.2% for precision, recall and F1 scores. compared to gold standard data extracted by two humans from a set of primary articles. A detailed error analysis revealed seven instances of confabulation events, representing 4% of the total extracted data. Given the comparable performance metrics between Elicit and ChatGPT, selection can be based on individual systematic review requirements and workflow preferences. We propose implementing AI‐assisted extraction in place of the second human data extraction leaving the second human data reviewer to compare and reconcile data extracted by one human and one AI tool.

## Code Availability Statement

All prompts used in the study have been made publicly available in Appendix 1.

## Author Contributions

**T. Helms Andersen:** conceptualization, investigation, writing – original draft, methodology, validation, visualization, writing – review and editing, software, formal analysis, project administration, data curation, supervision, resources. **T. M. Marcussen:** investigation, methodology, validation, visualization, writing – review and editing, writing – original draft, software, formal analysis, data curation, conceptualization. **A. D. Termannsen:** validation, investigation, writing – review and editing. **T. W. H. Lawaetz:** validation, investigation, writing – review and editing. **O. Nørgaard:** conceptualization, investigation, methodology, validation, writing – review and editing, writing – original draft, formal analysis, resources, software, supervision.

## Conflicts of Interest

The authors declare no conflicts of interest.

## 1 Peer Review

The peer review history for this article is available at https://www.webofscience.com/api/gateway/wos/peer-review/10.1002/cesm.70036.

## Supporting information

Andersen TH CESM DeclarationofInterest.

## Data Availability

The data that support the findings of this study are available from the corresponding author upon reasonable request.

## Appendix 1: Prompts

### Generic Prompts for Others to Use

#### Prompt 1 – Population Characteristics

You are an experienced researcher who masters the extraction of data from scientific articles of research studies. Your task is to review and extract data from a scientific article uploaded as a PDF. The data of interest is listed below under the heading ‘*Data to extract*’. Your response should be extremely comprehensive and strictly follow these rules:

– If there was a prompt before this one, disregard it and start over. Work with the PDF as if you are seeing it for the first time.
– Extract data only from the article's own results, not from cited sources in the introduction or discussion sections.
– Report only data relevant to the article's studied population.
– If data is not available, state: ‘Data could not be found.’
– Ensure the list includes only the elements verified as being researched, formatted as bullet points for clarity. Extract data verbatim whenever it is possible.
– After presenting data, assess and indicate your confidence in its ability to represent what is reported in the article by adding a confidence level (High, Medium, or Low) in parentheses. For example: ‘20,000 participants (High).’
– Continue searching for more relevant information even after finding initial data. Do not stop until you have thoroughly reviewed the entire article. Ensure all relevant data is reported.

Ensure your extraction is based directly on the article's data, accurately reflecting the information provided in the article.

Before finalizing your report, thoroughly compare all reported information with the original text to ensure accuracy. Triple check each detail to confirm it matches exactly.

There will be a penalty if you do not follow and adhere to all the instructions you have been given.

*Data to extract*

Population characteristics:

For [the (sub)population of interest], please list:

– Number of participants
– Age distribution of participants
– Sex distribution of participants
– [Review specific population characteristics, insert more as needed]

**Prompt 2 – Study design**

You are an experienced researcher who masters the extraction of data from scientific articles of research studies. Your task is to review and extract data from a scientific article uploaded as a PDF. The data of interest is listed below under the heading ‘*Data to extract*’. Your response should be extremely comprehensive and strictly follow these rules:

– If there was a prompt before this one, disregard it and start over. Work with the PDF as if you are seeing it for the first time.
– Extract data only from the article's own results, not from cited sources in the introduction or discussion sections.
– Report only data relevant to the article's studied population.
– If data is not available, state: ‘Data could not be found.’
– Ensure the list includes only the elements verified as being researched, formatted as bullet points for clarity. Extract data verbatim whenever it is possible.
– After presenting data, assess and indicate your confidence in its ability to represent what is reported in the article by adding a confidence level (High, Medium, or Low) in parentheses. For example: ‘20,000 participants (High).’
– Continue searching for more relevant information even after finding initial data,. Do not stop until you have thoroughly reviewed the entire article. Ensure all relevant data is reported.

Ensure your extraction is based directly on the article's data, accurately reflecting the information provided in the article.

Before finalizing your report, thoroughly compare all reported information with the original text to ensure accuracy. Triple check each detail to confirm it matches exactly.

There will be a penalty if you do not follow and adhere to all the instructions you have been given.

*Data to extract*

Study design:

Analyze the provided scientific article to determine the study design. Examine the abstract, methodology, and other relevant sections to identify whether it's a randomized controlled trial (RCT), cohort study, case‐control study, cross‐sectional study, qualitative study, or another type of study design. State nothing else than the design type.

**Prompt 3 – Review specific variable**

You are an experienced researcher who masters the extraction of data from scientific articles of research studies. Your task is to review and extract data from a scientific article uploaded as a PDF. The data of interest is listed below under the heading ‘*Data to extract*’. Your response should be extremely comprehensive and strictly follow these rules:

– If there was a prompt before this one, disregard it and start over. Work with the PDF as if you are seeing it for the first time.
– Extract data only from the article's own results, not from cited sources in the introduction or discussion sections.
– Report only data relevant to the article's studied population.
– If data is not available, state: ‘Data could not be found.’
– Ensure the list includes only the elements verified as being researched, formatted as bullet points for clarity. Extract data verbatim whenever it is possible.
– After presenting data, assess and indicate your confidence in its ability to represent what is reported in the article by adding a confidence level (High, Medium, or Low) in parentheses. For example: ‘20,000 participants (High).’
– Continue searching for more relevant information even after finding initial data. Do not stop until you have thoroughly reviewed the entire article. Ensure all relevant data is reported.

Ensure your extraction is based directly on the article's data, accurately reflecting the information provided in the article.

Before finalizing your report, thoroughly compare all reported information with the original text to ensure accuracy. Triple check each detail to confirm it matches exactly.

There will be a penalty if you do not follow and adhere to all the instructions you have been given.

*Data to extract*

[Review specific variable prompt]:

Provide a comprehensible unordered bullet point list of the following information, as text excerpts from the PDF:

[Review‐specific variable, insert what you need extracted]

### Prompts Used for Andersen et al.

#### Prompt 1

You are an experienced researcher who masters the extraction of data from scientific articles of research studies. Your task is to review and extract data from a scientific article uploaded as a PDF. The data of interest is listed below under the heading ‘*Data to extract*’. Your response should be extremely comprehensive and strictly follow these rules:

– If there was a prompt before this one, disregard it and start over. Work with the PDF as if you are seeing it for the first time.
– Extract data only from the article's own results, not from cited sources in the introduction or discussion sections.
– Report only data relevant to the article's studied population.
– If data is not available, state: ‘Data could not be found.’
– Ensure the list includes only the elements verified as being researched, formatted as bullet points for clarity. Extract data verbatim whenever it is possible.
– After presenting data, assess and indicate your confidence in its ability to represent what is reported in the article by adding a confidence level (High, Medium, or Low) in parentheses. For example: ‘20,000 participants (High).’
– Continue searching for more relevant information even after finding initial data. Do not stop until you have thoroughly reviewed the entire article. Ensure all relevant data is reported.

Ensure your extraction is based directly on the article's data, accurately reflecting the information provided in the article.

Before finalizing your report, thoroughly compare all reported information with the original text to ensure accuracy. Triple check each detail to confirm it matches exactly.

There will be a penalty if you do not follow and adhere to all the instructions you have been given.

*Data to extract*

Population characteristics:

For physicians, please list:

– Number of participants
– Age distribution of participants
– Sex distribution of participants
– Response rate
– Types and distribution of physicians
– Size of practice
– Years practiced

**Prompt 2**

You are an experienced researcher who masters the extraction of data from scientific articles of research studies. Your task is to review and extract data from a scientific article uploaded as a PDF. The data of interest is listed below under the heading ‘*Data to extract*’. Your response should be extremely comprehensive and strictly follow these rules:

– If there was a prompt before this one, disregard it and start over. Work with the PDF as if you are seeing it for the first time.
– Extract data only from the article's own results, not from cited sources in the introduction or discussion sections.
– Report only data relevant to the article's studied population.
– If data is not available, state: ‘Data could not be found.’
– Ensure the list includes only the elements verified as being researched, formatted as bullet points for clarity. Extract data verbatim whenever it is possible.
– After presenting data, assess and indicate your confidence in its ability to represent what is reported in the article by adding a confidence level (High, Medium, or Low) in parentheses. For example: ‘20,000 participants (High).’
– Continue searching for more relevant information even after finding initial data. Do not stop until you have thoroughly reviewed the entire article. Ensure all relevant data is reported.

Ensure your extraction is based directly on the article's data, accurately reflecting the information provided in the article.

Before finalizing your report, thoroughly compare all reported information with the original text to ensure accuracy. Triple check each detail to confirm it matches exactly.

There will be a penalty if you do not follow and adhere to all the instructions you have been given.

*Data to extract*

Study design:

Analyze the provided scientific article to determine the study design. Examine the abstract, methodology, and other relevant sections to identify whether it's a randomized controlled trial (RCT), cohort study, case‐control study, cross‐sectional study, qualitative study, or another type of study design. State nothing else than the design type.

**Prompt 3**

You are an experienced researcher who masters the extraction of data from scientific articles of research studies. Your task is to review and extract data from a scientific article uploaded as a PDF. The data of interest is listed below under the heading ‘*Data to extract*’. Your response should be extremely comprehensive and strictly follow these rules:

– If there was a prompt before this one, disregard it and start over. Work with the PDF as if you are seeing it for the first time.
– Extract data only from the article's own results, not from cited sources in the introduction or discussion sections.
– Report only data relevant to the article's studied population.
– If data is not available, state: ‘Data could not be found.’
– Ensure the list includes only the elements verified as being researched, formatted as bullet points for clarity. Extract data verbatim whenever it is possible.
– After presenting data, assess and indicate your confidence in its ability to represent what is reported in the article by adding a confidence level (High, Medium, or Low) in parentheses. For example: ‘20,000 participants (High).’
– Continue searching for more relevant information even after finding initial data. Do not stop until you have thoroughly reviewed the entire article. Ensure all relevant data is reported.

Ensure your extraction is based directly on the article's data, accurately reflecting the information provided in the article.

Before finalizing your report, thoroughly compare all reported information with the original text to ensure accuracy. Triple check each detail to confirm it matches exactly.

There will be a penalty if you do not follow and adhere to all the instructions you have been given.

*Data to extract*

Information needs:

Provide a comprehensible unordered bullet point list of the following information, as text excerpts from the PDF:

– information or knowledge needs for physicians regarding type‐2 diabetes.

### Prompts Used for Lawaetz et al.

#### Prompt 1

You are an experienced researcher who masters the extraction of data from scientific articles of research studies. Your task is to review and extract data from a scientific article uploaded as a PDF. The data of interest is listed below under the heading ‘*Data to extract*’. Your response should be extremely comprehensive and strictly follow these rules:

– If there was a prompt before this one, disregard it and start over. Work with the PDF as if you are seeing it for the first time.
– Extract data only from the article's own results, not from cited sources in the introduction or discussion sections.
– Report only data relevant to the article's studied population.
– If data is not available, state: ‘Data could not be found.’
– Ensure the list includes only the elements verified as being researched, formatted as bullet points for clarity. Extract data verbatim whenever it is possible.
– ‐After presenting data, assess and indicate your confidence in its ability to represent what is reported in the article by adding a confidence level (High, Medium, or Low) in parentheses. For example: ‘20,000 participants (High).’
– Continue searching for more relevant information even after finding initial data. Do not stop until you have thoroughly reviewed the entire article. Ensure all relevant data is reported.

Ensure your extraction is based directly on the article's data, accurately reflecting the information provided in the article.

Before finalizing your report, thoroughly compare all reported information with the original text to ensure accuracy. Triple check each detail to confirm it matches exactly.

There will be a penalty if you do not follow and adhere to all the instructions you have been given.

*Data to extract*

Population characteristics:

For both the control and the intervention population, please list:

– Number of participants
– Age distribution of participants
– Sex distribution of participants

**Prompt 2**

You are an experienced researcher who masters the extraction of data from scientific articles of research studies. Your task is to review and extract data from a scientific article uploaded as a PDF. The data of interest is listed below under the heading ‘*Data to extract*’. Your response should be extremely comprehensive and strictly follow these rules:

– If there was a prompt before this one, disregard it and start over. Work with the PDF as if you are seeing it for the first time.
– Extract data only from the article's own results, not from cited sources in the introduction or discussion sections.
– Report only data relevant to the article's studied population.
– If data is not available, state: ‘Data could not be found.’
– Ensure the list includes only the elements verified as being researched, formatted as bullet points for clarity. Extract data verbatim whenever it is possible.
– After presenting data, assess and indicate your confidence in its ability to represent what is reported in the article by adding a confidence level (High, Medium, or Low) in parentheses. For example: ‘20,000 participants (High).’
– Continue searching for more relevant information even after finding initial data. Do not stop until you have thoroughly reviewed the entire article. Ensure all relevant data is reported.

Ensure your extraction is based directly on the article's data, accurately reflecting the information provided in the article.

Before finalizing your report, thoroughly compare all reported information with the original text to ensure accuracy. Triple check each detail to confirm it matches exactly.

There will be a penalty if you do not follow and adhere to all the instructions you have been given.

*Data to extract*

Study design:

Analyze the provided scientific article to determine the study design. Examine the abstract, methodology, and other relevant sections to identify whether it's a randomized controlled trial (RCT), cohort study, case‐control study, cross‐sectional study, qualitative study, or another type of study design. State nothing else than the design type.

**Prompt 3**

You are an experienced researcher who masters the extraction of data from scientific articles of research studies. Your task is to review and extract data from a scientific article uploaded as a PDF. The data of interest is listed below under the heading ‘*Data to extract*’. Your response should be extremely comprehensive and strictly follow these rules:

– If there was a prompt before this one, disregard it and start over. Work with the PDF as if you are seeing it for the first time.
– Extract data only from the article's own results, not from cited sources in the introduction or discussion sections.
– Report only data relevant to the article's studied population.
– If data is not available, state: ‘Data could not be found.’
– Ensure the list includes only the elements verified as being researched, formatted as bullet points for clarity. Extract data verbatim whenever it is possible.
– After presenting data, assess and indicate your confidence in its ability to represent what is reported in the article by adding a confidence level (High, Medium, or Low) in parentheses. For example: ‘20,000 participants (High).’
– Continue searching for more relevant information even after finding initial data. Do not stop until you have thoroughly reviewed the entire article. Ensure all relevant data is reported.

Ensure your extraction is based directly on the article's data, accurately reflecting the information provided in the article.

Before finalizing your report, thoroughly compare all reported information with the original text to ensure accuracy. Triple check each detail to confirm it matches exactly.

There will be a penalty if you do not follow and adhere to all the instructions you have been given.

*Data to extract*

Incretins:

Provide a comprehensible unordered bullet point list of the following information, as text excerpts from the PDF:

– Information about GLP‐1 concentrations with units. State both means and standard deviations (sd). state if GLP‐1 is in active or total form. Also state if it is measured fasting or after an intervention, and provide a description of the studied group (e.g., 1 month post diagnosis).
– Information about GIP concentrations with units. State both means and standard deviations (sd). Also state if it is measured fasting or after an intervention, and provide a description of the studied group (e.g., 1 month post diagnosis).

### Prompts Used for Termannsen et al.

#### Prompt 1

You are an experienced researcher who masters the extraction of data from scientific articles of research studies. Your task is to review and extract data from a scientific article uploaded as a PDF. The data of interest is listed below under the heading ‘*Data to extract*’. Your response should be extremely comprehensive and strictly follow these rules:

– If there was a prompt before this one, disregard it and start over. Work with the PDF as if you are seeing it for the first time.
– Extract data only from the article's own results, not from cited sources in the introduction or discussion sections.
– Report only data relevant to the article's studied population.
– If data is not available, state: ‘Data could not be found.’
– Ensure the list includes only the elements verified as being researched, formatted as bullet points for clarity. Extract data verbatim whenever it is possible.
– After presenting data, assess and indicate your confidence in its ability to represent what is reported in the article by adding a confidence level (High, Medium, or Low) in parentheses. For example: ‘20,000 participants (High).’
– Continue searching for more relevant information even after finding initial data. Do not stop until you have thoroughly reviewed the entire article. Ensure all relevant data is reported.

Ensure your extraction is based directly on the article's data, accurately reflecting the information provided in the article.

Before finalizing your report, thoroughly compare all reported information with the original text to ensure accuracy. Triple check each detail to confirm it matches exactly.

There will be a penalty if you do not follow and adhere to all the instructions you have been given.

*Data to extract*

Population characteristics:

For both control and intervention groups, please list:

– Number of participants
– Age distribution of participants
– Sex distribution of participants

**Prompt 2**

You are an experienced researcher who masters the extraction of data from scientific articles of research studies. Your task is to review and extract data from a scientific article uploaded as a PDF. The data of interest is listed below under the heading ‘*Data to extract*’. Your response should be extremely comprehensive and strictly follow these rules:

– If there was a prompt before this one, disregard it and start over. Work with the PDF as if you are seeing it for the first time.
– Extract data only from the article's own results, not from cited sources in the introduction or discussion sections.
– Report only data relevant to the article's studied population.
– If data is not available, state: ‘Data could not be found.’
– Ensure the list includes only the elements verified as being researched, formatted as bullet points for clarity. Extract data verbatim whenever it is possible.
– After presenting data, assess and indicate your confidence in its ability to represent what is reported in the article by adding a confidence level (High, Medium, or Low) in parentheses. For example: ‘20,000 participants (High).’
– Continue searching for more relevant information even after finding initial data. Do not stop until you have thoroughly reviewed the entire article. Ensure all relevant data is reported.

Ensure your extraction is based directly on the article's data, accurately reflecting the information provided in the article.

Before finalizing your report, thoroughly compare all reported information with the original text to ensure accuracy. Triple check each detail to confirm it matches exactly.

There will be a penalty if you do not follow and adhere to all the instructions you have been given.

*Data to extract*

Study design:

Analyze the provided scientific article to determine the study design. Examine the abstract, methodology, and other relevant sections to identify whether it's a randomized controlled trial (RCT), cohort study, case‐control study, cross‐sectional study, qualitative study, or another type of study design. State nothing else than the design type.

**Prompt 3**

You are an experienced researcher who masters the extraction of data from scientific articles of research studies. Your task is to review and extract data from a scientific article uploaded as a PDF. The data of interest is listed below under the heading ‘*Data to extract*’. Your response should be extremely comprehensive and strictly follow these rules:

– If there was a prompt before this one, disregard it and start over. Work with the PDF as if you are seeing it for the first time.
– Extract data only from the article's own results, not from cited sources in the introduction or discussion sections.
– Report only data relevant to the article's studied population.
– If data is not available, state: ‘Data could not be found.’
– Ensure the list includes only the elements verified as being researched, formatted as bullet points for clarity. Extract data verbatim whenever it is possible.
– After presenting data, assess and indicate your confidence in its ability to represent what is reported in the article by adding a confidence level (High, Medium, or Low) in parentheses. For example: ‘20,000 participants (High).’
– Continue searching for more relevant information even after finding initial data. Do not stop until you have thoroughly reviewed the entire article. Ensure all relevant data is reported.

Ensure your extraction is based directly on the article's data, accurately reflecting the information provided in the article.

Before finalizing your report, thoroughly compare all reported information with the original text to ensure accuracy. Triple check each detail to confirm it matches exactly.

There will be a penalty if you do not follow and adhere to all the instructions you have been given.

*Data to extract*

Bodyweight and BMI:

Provide a comprehensible unordered bullet point list of the following information, as text excerpts from the PDF:

– Data on bodyweight for intervention group at baseline and post intervention, including mean and variance
– Data on bodyweight for control group at baseline and post intervention, including mean and variance
– Data on BMI for intervention group at baseline and post intervention, including mean and variance
– Data on BMI for control group at baseline and post intervention, including mean and variance.

## Appendix 2 Compared articles

**From Andersen et al.**

1. El‐Beheiry M, Vergis A, Choi JU, Clouston K, Hardy K. A survey of primary care physician referral to bariatric surgery in Manitoba: access, perceptions and barriers. Ann Transl Med. 2020 Mar;8(S1):S3.
2. Chu L, Bhogal SK, Lin P, Steele A, Fuller M, Ciaccia A, et al. AWAREness of Diagnosis and Treatment of Chronic Kidney Disease in Adults With Type 2 Diabetes (AWARE‐CKD in T2D). Canadian Journal of Diabetes. 2022 Jul;46(5):464–72.
3. Williamson JC, Glauser TA, Nevins PH, Schneider D, Kruger DF, Urquhart BS, et al. Current Practice Patterns and Identified Educational Needs of Health Care Providers in Managing Patients With Type 2 Diabetes. Clinical Diabetes. 2013 Jan 1;31(1):3–9.
4. Rubin DJ, Moshang J, Jabbour SA. Diabetes knowledge: Are resident physicians and Nurses adequately prepared to manage diabetes? Endocrine Practice. 2007 Jan;13(1):17–21.
5. Shahla L, Vasudev R, Chitturi C, Rodriguez C, Paul N. Diabetes mellitus treatment—Related medical knowledge among health care providers. Diabetes & Metabolic Syndrome: Clinical Research & Reviews. 2017 Jan;11(1):69–72.
6. Rayanagoudar G, Moore M, Zamora J, Hanson P, Huda MSB, Hitman GA, et al. Postpartum care of women with gestational diabetes: survey of healthcare professionals. European Journal of Obstetrics & Gynecology and Reproductive Biology. 2015 Nov;194:236–40.
7. Shubrook JH, Pak J, Dailey G. Primary Care Physicians’ Knowledge of the Cardiovascular Effects of Diabetes Medications: Findings from an Online Survey. 2020 [cited 2025 Jan 20];14189465 Bytes.
8. Wiggins MN, Landes RD, Bhaleeya SD, Uwaydat SH. Primary care physicians’ knowledge of the ophthalmic effects of diabetes. Canadian Journal of Ophthalmology. 2013 Aug;48(4):265–8.
9. George J, Hannah S, Lang CC. ThiaZolidineDiones and the Influence of Media Adverse Reporting on Prescribing Attitudes in PraCTice (TZD‐IMPACT) Study. Cardiovascular Therapeutics. 2009 Jun;27(2):83–8.
10. Özgüç H, Narmanlı M, Çırnaz H. Turkish primary care physicians’ attitudes and knowledge of obesity and bariatric surgery: a survey study. Turk J Surg. 2021 Sep 1;37(3):266–76.

**From Lawaetz et al.**

1. Tell SS, Schafer M, Vigers T, Baumgartner AD, Lyon E, Gross S, et al. Bromocriptine quick‐release as adjunct therapy in youth and adults with type 1 diabetes: A randomized, placebo‐controlled crossover study. Diabetes Obesity Metabolism. 2022 Nov;24(11):2148–58.
2. Ho J, Nicolucci AC, Virtanen H, Schick A, Meddings J, Reimer RA, et al. Effect of Prebiotic on Microbiota, Intestinal Permeability, and Glycemic Control in Children With Type 1 Diabetes. The Journal of Clinical Endocrinology & Metabolism. 2019 Oct 1;104(10):4427–40.
3. Harray AJ, Binkowski S, Keating BL, Horowitz M, Standfield S, Smith G, et al. Effects of Dietary Fat and Protein on Glucoregulatory Hormones in Adolescents and Young Adults With Type 1 Diabetes. The Journal of Clinical Endocrinology & Metabolism. 2022 Jan 1;107(1):e205–13.
4. Lodefalk M, Carlsson‐Skwirut C, Holst JJ, Åman J, Bang P. Effects of Fat Supplementation on Postprandial GIP, GLP‐1, Ghrelin and IGFBP‐1 Levels: A Pilot Study on Adolescents with Type 1 Diabetes. Horm Res Paediatr. 2010;73(5):355–62.
5. Akıncı A, Aydın Ö, Özerol Hİ. Glucagon‐like Peptide‐1 and‐2 Levels in Children with Diabetic Ketoacidosis. jcrpe. 2011 Jan 10;1(3):144–50.
6. Huml M, Kobr J, Siala K, Varvařovská J, Pomahačová R, Karlíková M, et al. Gut Peptide Hormones and Pediatric Type 1 Diabetes Mellitus. Physiol Res. 2011 Aug 31;647–58.
7. Pörksen S, Nielsen LB, Kaas A, Kocova M, Chiarelli F, Ørskov C, et al. Meal‐Stimulated Glucagon Release Is Associated with Postprandial Blood Glucose Level and Does Not Interfere with Glycemic Control in Children and Adolescents with New‐Onset Type 1 Diabetes. The Journal of Clinical Endocrinology & Metabolism. 2007 Aug 1;92(8):2910–6.
8. Kaas A, Max Andersen ML, Fredheim S, Hougaard P, Buschard K, Petersen JS, et al. Proinsulin, GLP‐1, and glucagon are associated with partial remission in children and adolescents with newly diagnosed type 1 diabetes. Pediatric Diabetes. 2012 Feb;13(1):51–8.
9. Fredheim S, Andersen MLM, Pörksen S, Nielsen LB, Pipper C, Hansen L, et al. The influence of glucagon on postprandial hyperglycaemia in children 5 years after onset of type 1 diabetes. Diabetologia. 2015 Apr;58(4):828–34.
10. Uslu B, Gurbuz F, Temiz F, Yuksel B, Mungan N, Topaloglu A. The Investigation of Plasma Glucagon‐like Peptide‐1 (glp‐1) Levels in Newly Diagnosed Type 1 Diabetic Children. West Indian Med J. 2016 Nov;65(1):141‐6.

**From Termannsen et al.**

1. Bunner AE, Wells CL, Gonzales J, Agarwal U, Bayat E, Barnard ND. A dietary intervention for chronic diabetic neuropathy pain: a randomized controlled pilot study. Nutr & Diabetes. 2015 May 26;5(5):e158.
2. Barnard ND, Cohen J, Jenkins DJA, Turner‐McGrievy G, Gloede L, Jaster B, et al. A Low‐Fat Vegan Diet Improves Glycemic Control and Cardiovascular Risk Factors in a Randomized Clinical Trial in Individuals With Type 2 Diabetes. Diabetes Care. 2006 Aug 1;29(8):1777–83.
3. Barnard ND, Alwarith J, Rembert E, Brandon L, Nguyen M, Goergen A, et al. A Mediterranean Diet and Low‐Fat Vegan Diet to Improve Body Weight and Cardiometabolic Risk Factors: A Randomized, Cross‐over Trial. Journal of the American Nutrition Association. 2022 Feb 17;41(2):127–39.
4. Turner‐McGrievy GM, Davidson CR, Wingard EE, Wilcox S, Frongillo EA. Comparative effectiveness of plant‐based diets for weight loss: A randomized controlled trial of five different diets. Nutrition. 2015 Feb;31(2):350–8.
5. Jenkins DJA, Wong JMW, Kendall CWC, Esfahani A, Ng VWY, Leong TCK, et al. Effect of a 6‐month vegan low‐carbohydrate (‘Eco‐Atkins’) diet on cardiovascular risk factors and body weight in hyperlipidaemic adults: a randomised controlled trial. BMJ Open. 2014 Feb;4(2):e003505.
6. Lee YM, Kim SA, Lee IK, Kim JG, Park KG, Jeong JY, et al. Effect of a Brown Rice Based Vegan Diet and Conventional Diabetic Diet on Glycemic Control of Patients with Type 2 Diabetes: A 12‐Week Randomized Clinical Trial. Meyre D, editor. PLoS ONE. 2016 Jun 2;11(6):e0155918.
7. Kahleova H, Petersen KF, Shulman GI, Alwarith J, Rembert E, Tura A, et al. Effect of a Low‐Fat Vegan Diet on Body Weight, Insulin Sensitivity, Postprandial Metabolism, and Intramyocellular and Hepatocellular Lipid Levels in Overweight Adults: A Randomized Clinical Trial. JAMA Netw Open. 2020 Nov 30;3(11):e2025454.
8. Wright N, Wilson L, Smith M, Duncan B, McHugh P. The BROAD study: A randomised controlled trial using a whole food plant‐based diet in the community for obesity, ischaemic heart disease or diabetes. Nutr & Diabetes. 2017 Mar 20;7(3):e256.
9. Barnard ND, Scialli AR, Turner‐McGrievy G, Lanou AJ, Glass J. The effects of a low‐fat, plant‐based dietary intervention on body weight, metabolism, and insulin sensitivity. The American Journal of Medicine. 2005 Sep;118(9):991–7.
10. Barnard ND, Levin SM, Gloede L, Flores R. Turning the Waiting Room into a Classroom: Weekly Classes Using a Vegan or a Portion‐Controlled Eating Plan Improve Diabetes Control in a Randomized Translational Study. Journal of the Academy of Nutrition and Dietetics. 2018 Jun;118(6):1072–9.
