# Supplementary material for: Using Artificial Intelligence Tools as Second Reviewers for Data Extraction in Systematic Reviews: A Performance Comparison of Two AI Tools Against Human Reviewers
Source: Cochrane Evid Synth Methods. 2025 Jul 14;3(4):e70036. doi: 10.1002/cesm.70036 (PMC12257877; doi:10.1002/cesm.70036)
Supplement: Supplementary file 1 — Andersen TH CESM DeclarationofInterest. [file CESM-3-e70036-s001.pdf]

CESM: Declaration of Interest

Cochrane Evidence Synthesis and Methods, as a Cochrane Collaboration journal abides by [Cochrane's conflict of interest policy for Cochrane Library content (2020)], which applies to all journal content. Cochrane's conflicts of interest policy for *Cochrane Evidence Synthesis and Methods* not only requires study funding and author interests to be declared at the earliest point possible, but also mandates that some funding and conflicts of interest will prevent people from being authors of submissions.

To support your submission, please confirm the following:

☒ Submitted content is not directly funded or produced by any commercial organization with a financial interest in the topic.

☒ Funders of submitted content are declared in the 'Acknowledgements' section of the manuscript, including a statement that the funder had no role in the design, conduct, or publication of the content, research, or evidence synthesis.

☒ All financial and non-financial interests within 36 months of the submission date have been declared by all authors at the time of submission.

☒ None of the authors has a direct financial interest, within 36 months of the submission date, in the topic of a submission.

☒ Authors without conflicts of interest make up at least two-thirds of the author team.

☒ First and last authors are entirely free of financial conflicts of interest.

☒ Neither the first or last author of the submitted content are authors of clinical studies that are funded by industry and are relevant to the topic of the submission.
